# Mycophenolate mofetil–induced hyperlipidemia with cutaneous manifestations

**DOI:** 10.1002/ccr3.7056

**Published:** 2023-03-14

**Authors:** Rebecca M. Yim, Vikram N. Sahni, Jason G. Mathis

**Affiliations:** ^1^ Tulane University School of Medicine New Orleans Louisiana USA; ^2^ Department of Dermatology University of Utah Salt Lake City Utah USA

**Keywords:** dermatology, hyperlipidemia, patient education < social care, pharmacology, “

## Abstract

We report a case of a 49 year old woman who developed biopsy‐proven xanthomas on her hands and arms after initiation of Mycophenolate mofetil therapy for systemic lupus erythematosus (SLE) and subsequently went into remission upon cessation of the medication.

## Abstract 2

It is necessary to evaluate the full side‐effect profile of systemic medications such as mycophenolate mofetil and the impact of this medication on lipid abnormalities. Xanthomas are a sparsely reported cutaneous manifestation of mycophenolate mofetil–induced dyslipidemia.

## 1 INTRODUCTION

Xanthomas are lipid deposits in the connective tissue of the skin, tendons, or fasciae that are predominantly composed of foam cells, which are macrophages that have excessively phagocytized recently oxidized low‐density lipoprotein cholesterol (LDL‐C) particles.^1^ Clinically, they are expressed as yellow‐orange papules, plaques, or nodules. Their clinical presentation requires that the differential diagnosis includes cutaneous tumors. However, xanthomas are not neoplasms or cutaneous tumors.^2^ These lesions represent an abnormal lipid transportation system in blood vessels and are associated with an increased risk of cardiovascular disease.^3^ Therefore, xanthoma manifestation is a significant symptom that requires careful investigation to uncover the underlying etiology.

While not always the case, xanthomas are often due to an underlying primary or secondary hyperlipidemia. Examples of primary hyperlipidemia include the manifestation of xanthoma tuberosum and tendinosum in people with familial hypercholesterolemia or palmar crease xanthoma in familial dysbetalipoproteinemia.^4^ Examples of secondary hyperlipidemia include underlying drug interactions or diseases that affect lipid metabolism such as hypothyroidism, nephrotic syndrome, and primary biliary cholangitis.

In drug‐induced hyperlipidemia, large increases in total cholesterol (TC), LDL‐C, and triglycerides of up to 40%, 50%, and 300%, respectively, have been reported.^5^ Drugs that are well known to cause secondary hyperlipidemia include diuretics, beta‐blocking agents, progestogens, combined oral contraceptives containing “second generation” progestogens, danazol, immunosuppressant agents, protease inhibitors, and enzyme‐inducing anticonvulsants.^5^ Immunosuppressant drugs that have an increased risk of inducing hyperlipidemia include cyclosporin, azathioprine, and corticosteroids with an increase in TC by 10%–40%, LDL‐C by 0%–50%, HDL‐C by 0%–90%, and triglycerides by 0%–70%.^5^ Previous reports indicated that mycophenolate mofetil does not cause adverse effects on lipid metabolism,^5^, ^6^ but more recent articles suggest that there is an association between mycophenolate mofetil and hyperlipidemia.^7^, ^8^ We report a case of a 49‐year‐old woman who developed biopsy‐proven xanthomas on her hands and arms after initiation of mycophenolate mofetil therapy for systemic lupus erythematosus (SLE).

## 2 CASE REPORT

The patient is a 49‐year‐old female with a pertinent medical history of SLE who presented for evaluation of painful, non‐pruritic, oozing lesions on her hands and arms. The patient denied any history of prior, similar lesions and noted that they first started to develop within days of initiation of mycophenolate mofetil therapy for control of her SLE.

They were described as indurated pink crusted papules, resembling hypertrophic lupus or small keratoacanthomas. A biopsy of a lesion showed focal epidermal spongiosis. Within the reticular dermis, there were numerous histiocytes with foamy cytoplasm interspersed by an infiltrate of lymphocytes with scattered neutrophils and rare eosinophils. Infectious etiology was ruled out with periodic acid‐Schiff (PASF), tissue gram stain, and acid‐fast bacterial culture failing to demonstrate fungal, bacterial, or mycobacterial forms. The diagnosis of eruptive xanthomas was made given the clinical history and pathologic correlation. The patient denied any familial history of heart disease, high cholesterol, or hyperlipidemia and was not on estrogen replacement therapy or any medications commonly associated with metabolic syndrome including systemic retinoids and antipsychotics.^5^

A lipid panel revealed normal triglycerides (92), HDL (51), and elevated LDL (150).

Management of this patient included cessation of mycophenolate mofetil and initiation of a statin, which resolved both the abnormal LDL level and cutaneous manifestations of disease. The patient has not had any relapses in condition even after stopping simvastatin.

## 3 DISCUSSION

A variety of medications have been associated with drug‐induced hyperlipidemia, including immunosuppressant drugs such as mycophenolate mofetil. The mechanism behind drug‐induced hypercholesterolemia has yet to be elucidated. Nuclear receptor pregnane X receptor (PXR; NR1I2), a ligand‐activated transcription factor primarily located in the liver and intestines, may play a role.^7^

PXR activation has been shown to regulate many phase 1 and 2 drug‐metabolizing enzymes and transporters, including cytochrome P450 (CYP) 3A4.^7^ PXR is a unique nuclear receptor because it has a large selection of structurally diverse ligands that can activate this receptor, thereby affecting not only drug metabolism, but also processes that aim to maintain physiologic homeostasis such as inflammation, cellular proliferation, and importantly, glucose and lipid metabolism.^7^ PXR has been shown to have adverse effects on glucose and lipid metabolism and blood pressure.^7^ PXR agonists increase TC and LDL‐C in the serum via CYP3A4 induction and increase endogenous cholesterol synthesis via increased rate of incorporation of C‐acetate into plasma cholesterol.^7^

The association between mycophenolate mofetil and hyperlipidemia has been reported in recent years. In cardiac transplant patients on immunosuppressant therapy receiving 3 g/day of mycophenolate mofetil, hypercholesterolemia was present in 41% of the patients.^8^ However, it was unclear as to what extent this metabolic effect was due to other immunosuppressants co‐administered with mycophenolate mofetil. Therefore, specific demonstration of mycophenolate mofetil–induced hyperlipidemia with sufficient deposition to cause xanthoma manifestations is a clinical rarity.

A possible explanation for mycophenolate mofetil–induced hyperlipidemia is PXR agonism.^7^ One study found that mycophenolic acid (MPA), the active parent drug of mycophenolate mofetil via in vivo enzymatic activity, is a PXR activator that was observed to enhance CYP3A4 expression,^9^ potentially increasing TC levels.^7^ An additional explanation could be due to its anti‐inflammatory effect. States of inflammation including infection decrease LDL‐C.^7^ Mycophenolate mofetil has anti‐inflammatory effects,^10^ so a resulting rise in LDL‐C levels would not be unexpected. Future studies should continue to further define the metabolic effect that mycophenolate mofetil has when administered both individually and in conjunction with other immunosuppressant medications.

In conclusion, mycophenolate mofetil can induce hyperlipidemia and cutaneous manifestations of disease including xanthomas. Although there are limited data proving its adverse effect on lipid metabolism, this case continues to promote the significant association between mycophenolate mofetil and hyperlipidemia. While the exact mechanism is unclear, mycophenolate mofetil has shown the ability to increase TC levels possibly via its anti‐inflammatory effects and MPA's PXR agonism.^7^ Further investigation is necessary to better elucidate the benefit–risk safety profile of mycophenolate mofetil and to caution patients who may have underlying dyslipidemia and increased cardiovascular risk.

## AUTHOR CONTRIBUTIONS

**Rebecca M Yim:** Conceptualization; writing – original draft; writing – review and editing. **Vikram Sahni:** Writing – original draft; writing – review and editing. **Jason Mathis:** Conceptualization; investigation; project administration; writing – review and editing.

## FUNDING INFORMATION

None.

## CONFLICT OF INTEREST STATEMENT

No conflicts of interest have been declared by the authors.

## CONSENT

Written informed consent was obtained from the patient to publish this report in accordance with the journal's patient consent policy.

## Data Availability

Data sharing not applicable ‐ no new data generated, or the article describes entirely theoretical research
